# Impact of Simulated Gastrointestinal Digestion on Antiglycoxidant Activity of Lemon Verbena (*Aloysia triphylla*) Herbal Tea and Characterization of Key Polyphenols via DPPH/MGO Pre-Column HPLC

**DOI:** 10.3390/antiox15060717

**Published:** 2026-06-05

**Authors:** Didier Fraisse, Alexis Bred, Catherine Felgines

**Affiliations:** Unité de Nutrition Humaine (UNH), CRNH Auvergne, INRAE, Université Clermont-Auvergne, F-63000 Clermont-Ferrand, France; alexis.bred@uca.fr (A.B.); catherine.felgines@uca.fr (C.F.)

**Keywords:** *Aloysia triphylla*, infusion, in vitro digestion, antioxidant, glycation, bioaccessibility

## Abstract

*Aloysia triphylla* (lemon verbena, LV) herbal tea is a rich source of phenolic compounds with recognized antioxidant and antiglycoxidant properties, although their stability during digestion remains insufficiently understood. This study evaluated the impact of simulated gastrointestinal digestion on the phenolic composition and bioactivity of LV infusion using a standardized *in vitro* model. Total phenolic, flavonoid, and phenolic acid contents were determined spectrophotometrically, while individual compounds were analyzed by HPLC. Antioxidant activity was assessed using complementary assays (DPPH•, ABTS•, FRAP, ORAC, and nitric oxide scavenging), and antiglycation activity was evaluated using a BSA/D-ribose model. Digestion did not significantly affect total phenolic and phenolic acid contents, whereas flavonoids moderately decreased during the intestinal phase. Verbascoside underwent partial degradation, leading to increased levels of isoverbascoside and caffeic acid. Despite these transformations, antioxidant capacity was maintained or enhanced, particularly in ABTS and ORAC assays, suggesting a contribution of digestion-derived metabolites. Antiglycation activity remained stable after digestion. Pre-column HPLC analyses identified verbascoside and its derivatives as the main contributors to radical scavenging and methylglyoxal trapping activities. These findings indicate that LV infusion retains its bioactive potential after digestion and supports its relevance as a functional beverage.

## 1. Introduction

Medicinal and aromatic plants have played a vital role in traditional medicine for centuries, providing natural remedies for various human ailments. In recent years, growing interest in their biological properties has led to extensive research exploring their therapeutic potential, bioactive compounds, and possible synergistic effects [1].

Herbal teas are appreciated not only for their pleasant flavors but also for their health benefits. They are rich in polyphenols, and many of their recognized properties are attributed to these phenolic compounds [2]. Beyond their antioxidant properties, polyphenols, particularly phenolic acids and flavonoids, can inhibit the formation of advanced glycation end products (AGEs) through various mechanisms, including suppression of free radical production, inhibition of methylglyoxal formation, and metal ion chelation [3].

*Aloysia triphylla*, commonly known as lemon verbena (LV), is a herbaceous plant from the Verbenaceae family. Native to South America, it is also cultivated in the Mediterranean region, and its leaves are widely used as herbal tea. Traditionally, LV infusions were consumed to relieve gastrointestinal disorders (flatulence, colic, indigestion, etc.), insomnia, fever, colds, asthma but also for their aromatic properties [4,5,6].

During the infusion process, only most polar phenolic compounds, such as flavone diglucuronides (e.g., luteolin 7-O-diglucuronide: L7DG) and phenylpropanoid glycosides (notably verbascoside, often cited as a major compound in LV tea), are extracted [7,8]. Verbascoside, also known as acteoside, is a polyphenol with strong antioxidant properties. Its chemical structure consists of hydroxyphenylethyl and cinnamic acid moieties linked to a β-glucopyranose via a glycosidic bond. The presence of two catechol groups in its structure contributes to its antioxidant activity [9]. A structure–activity relationship study [9] further demonstrated the significant antioxidant potential of verbascoside and its isomer, isoverbascoside. Additionally, flavonoids such L7DG also exhibit remarkable antioxidant activity [10].

However, despite the well-documented antioxidant and anti-inflammatory properties of phenolic compounds, it remains unclear how these properties are preserved after digestion. To better understand these aspects, various in vitro digestion models have been developed to simulate gastrointestinal conditions [11]. These models are widely used to assess the bioaccessibility of nutrients, particularly polyphenols [12,13]. In vitro digestion studies investigating the stability, bioaccessibility, and bioavailability of verbascoside and isoverbascoside have previously been reported by Cardinali et al. [14]. However, to date, no in vitro digestion data are available regarding the digestive stability and antiglycoxidant activity of the phenolic compounds present in lemon verbena herbal tea.

Therefore, this study aimed to evaluate the impact of the digestion process on the phenolic composition of LV tea using a simulated in vitro digestion model, and to investigate its antioxidant and antiglycation activities.

## 2. Materials and Methods

### 2.1. Plant Material and Reagents

LV (*Aloysia triphylla*) leaves were from “Les 2 Marmottes,” a French herbal tea producer (Bons-en-Chablais, France) and were pooled from 4 batches (FB221213, FB221019, FB230326A, FB220311C). Analytical standards including verbascoside and isoverbascoside (HPLC grade, 99.0% purity), caffeic acid (HPLC grade, 99.0% purity), and L7DG (94.0% purity) were from Extrasynthese (Genay, France). Gallic acid (99.0% purity) and Trolox (6-hydroxy-2,5,7,8-tetramethylchroman-2-carboxylic acid, 97.0% purity) were obtained from Sigma-Aldrich Chemicals (Saint-Quentin Fallavier, France). Chromatographic-grade ethanol, methanol, and acetonitrile were supplied by Carlo Erba Reagents SAS (Val-de-Reuil, France), and phosphoric acid (85.0%), hydrochloric acid (37.0% *w*/*w*), and sodium hydroxide were purchased from VWR Prolabo (Fontenay-sous-Bois, France). All other chemicals were from Sigma-Aldrich. All aqueous solutions were prepared using ultrapure water (18.2 MΩ·cm) generated by a Milli-Q system (Merck, Darmstadt, Germany).

### 2.2. Infusion Preparation

Infusions are usually prepared using 10–50 g/L of dried plant material. To obtain usable analytical signals in this polyphenol-rich matrix, 5.0 g of dried LV leaves was infused in 100 mL of boiling deionized water for 30 min. The infusion was then filtered and used for the in vitro digestion experiments.

### 2.3. In Vitro Simulated Gastrointestinal Digestion Procedure

Various digestion models have been developed, often hindering the possibility of comparing results across research teams. Our in vitro simulated gastrointestinal digestion (SGID) was conducted following the standardized method described by Minekus et al. (2014) [11], that has reached consensus within the COST Infogest network. This method is based on physiologically relevant conditions, applicable to different evaluation criteria, and can be modified to meet specific requirements.

**Figure 1:**
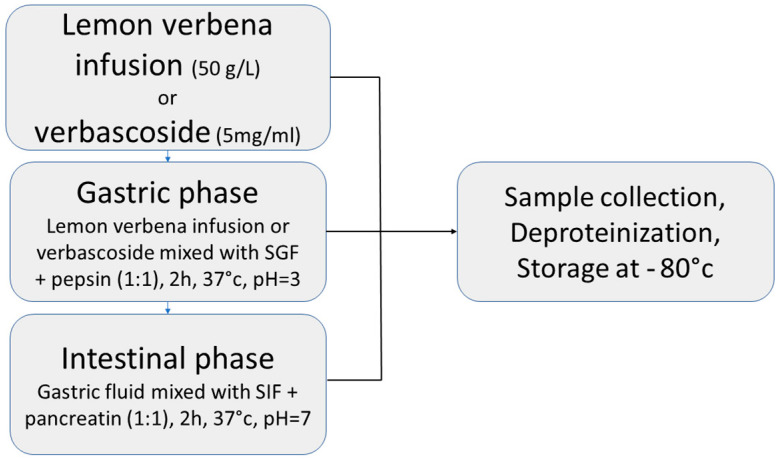
Flow diagram illustrating the in vitro simulated gastrointestinal digestion protocol. SGF: Simulated Gastric Fluid, SIF: Simulated Intestinal Fluid.

Since our work focused on a herbal tea, we did not consider it necessary to include the salivary phase, but focused only on the gastric and intestinal phases (Figure 1).

Simulated Gastric Fluid (SGF) and Simulated Intestinal Fluid (SIF) stock solutions were prepared according to Minekus et al. (2014) [11] and diluted similarly during the digestion process. All experiments were performed in triplicate (*n* = 3). Simulation of the gastric and intestinal phases followed the approach previously described [15].

During the gastric phase, 5 mL of LV herbal tea (50 g/L) was mixed with 3 mL of SGF stock solution and 1 mL of pepsin solution (20,000 U/mL in SGF). Calcium chloride (0.075 mM in the final gastric medium) and water were added to achieve a total volume of 10 mL, and the pH was adjusted to 3.0 using 1 M HCl. This gastric mixture was incubated at 37 °C for two hours with continuous agitation at 50 rpm in an orbital shaking incubator (NB-205 L, N-Biotek, Bucheon-si, Republic of Korea). Subsequently, 5 mL of the gastric digest was used for the intestinal phase, while the remaining 5 mL was reserved for chemical and biological analyses.

For the intestinal phase, 5 mL of the gastric digest was combined with 3 mL of SIF stock solution and 1 mL of pancreatin solution (1000 U/mL in SIF). The final volume was adjusted to 10 mL by adding calcium chloride (to obtain 0.3 mM in the final intestinal medium) and purified water, and the pH was set to 7.0 using 0.1 M NaOH. The mixture was then incubated for an additional two hours in an orbital shaking incubator under the same conditions as before (37 °C, 50 rpm).

To normalize LV tea concentrations between the gastric and intestinal matrices, gastric samples were diluted twofold with purified water. All samples were immediately deproteinized by adding four volumes of ethanol, followed by centrifugation for 15 min at 4300 rpm (Centrifuge 5804 R, Eppendorf, Montesson, France). Supernatants were aliquoted into 1 mL fractions and stored at −80 °C until analysis. Undigested LV tea was diluted fourfold with purified water and subjected to the same deproteinization, centrifugation, and storage procedure as the digested samples, to serve as a reference.

SGID of verbascoside (initial concentration: 5 mg/mL H_2_O) was performed under the same conditions.

### 2.4. Determination of Total Phenolic (TPC), Total Flavonoid (TFC), and Total Phenolic Acid (TPAC) Contents

Total Phenolic Content (TPC) was measured using a previously reported method [7]. LV infusion and verbascoside samples (undigested, gastric or intestinal) were diluted twofold in purified water, and 20 µL of these diluted solutions was mixed with 10 µL of undiluted Folin–Ciocalteu reagent and 100 µL of water. The final volume was adjusted to 250 µL using a sodium carbonate solution (150 g/L). After 30 min of incubation at room temperature, absorbance was recorded at 740 nm using a TECAN Infinite F200 PRO microplate reader (TECAN, Lyon, France). TPC results were expressed as milligrams of gallic acid equivalents (GAE) per gram of dry leaves, based on a gallic acid standard curve (0.0125–0.2 mg/mL, R^2^ = 0.9999, y = 287.05x + 0.1012).

Total Flavonoid Content (TFC) was determined using a modified aluminum chloride (AlCl_3_) spectrophotometric method [16]. Equal volumes of LV digestion samples (undigested, gastric, or intestinal) and 1% AlCl_3_·6H_2_O in methanol were mixed and incubated for 10 min at room temperature. Absorbance was measured at 430 nm using a Jasco V-630 spectrophotometer (Jasco, Lisses, France), and results were expressed as milligrams of L7DG equivalents (L7DGE) per gram of dry leaves, based on a standard curve (0.0095–0.3 mg/mL, R^2^ = 0.9985, y = 6.0942x + 0.0344).

Total Phenolic Acid Content (TPAC) was quantified using a modified Arnow method [17]. Diluted LV digestion samples (250 μL) were mixed with 1500 μL of water, 250 μL of 0.5 M HCl, 250 μL of Arnow reagent, and 250 μL of 1 M NaOH. Absorbance was measured immediately at 505 nm using a Jasco V-630 spectrophotometer. Results were expressed as milligrams of verbascoside equivalents (VE) per gram of dry leaves, based on a standard curve of verbascoside (0.0625–1 mg/mL, R^2^ = 0.9781, y = 4.2237x + 0.0481).

### 2.5. Evaluation of the Antioxidant Capacity

#### 2.5.1. DPPH• Radical Scavenging Assay

The DPPH• (2,2-diphenyl-1-picrylhydrazyl) scavenging activity was assessed following the protocol previously described [18]. In summary, 50 µL of digested and undigested solutions was combined with 2.5 mL of a freshly prepared DPPH• radical solution (25 µg/mL in methanol). The mixture was incubated at room temperature for 30 min, after which the absorbance was recorded at 515 nm using a Jasco V-630 UV-Vis spectrophotometer. The scavenging capacity of DPPH• was quantified as micromoles of Trolox equivalent (µmol TE) per gram of dry leaves or commercial standard, calculated using a standard Trolox calibration curve (100–3000 µmol/L, R^2^ = 0.997, y = 1101.3x + 1.521).

#### 2.5.2. Trolox Equivalent Antioxidant Capacity (TEAC) Assay

The ABTS•+ (2,2′-azino-bis(3-ethylbenzothiazoline-6-sulfonic acid) diammonium salt) scavenging capacity was evaluated as previously reported [15]. Specifically, 10 µL of digested and undigested solutions, diluted 2-fold with water, were combined with 250 μL of a freshly prepared ABTS•+ solution. After a 10 min incubation at room temperature, the absorbance was measured at 734 nm using a TECAN Infinite F200 PRO microplate reader. The ABTS•+ scavenging activity was calculated as micromoles of Trolox equivalent (µmol TE) per gram of dry sample or commercial standard, using a Trolox standard curve (75–300 µmol/L, R^2^ = 0.9993, y = 11.761x + 0.5494).

#### 2.5.3. Ferric Reducing Antioxidant Power (FRAP) Assay

The FRAP assay was conducted following the method of Katalinić et al. [19] with slight modifications. The stock solutions included a 300 mM acetate buffer (prepared with 3.1 g of C_2_H_3_NaO_2_·3H_2_O and 16 mL of acetic acid) at pH 3.6, a 10 mM TPTZ (2,4,6-tripyridyl-s-triazine) solution in 40 mM HCl, and a 20 mM FeCl_3_·6H_2_O solution. A fresh FRAP working solution was prepared by combining 25 mL of acetate buffer, 2.5 mL of TPTZ solution, and 2.5 mL of FeCl_3_·6H_2_O solution. A 10 μL aliquot of herbal tea samples (undigested, gastric, or intestinal) was mixed with 200 μL of FRAP solution. After 30 min incubation at room temperature in the dark, the absorbance of the ferrous complex was measured at 593 nm using a TECAN Infinite F200 PRO microplate reader. The results were expressed as µmol TE per gram of sample, based on a Trolox standard curve (50–750 µmol/L; R^2^ = 0.9147, y = 13,523x + 28.58).

#### 2.5.4. Oxygen Radical Absorbance Capacity (ORAC) Assay

The ability of the samples to scavenge peroxyl radicals was evaluated using the ORAC method, as previously described [20]. In total, 25 μL of herbal tea samples (undigested, gastric, or intestinal) was incubated with 150 μL of fluorescein solution (0.2 μM in phosphate buffer, 75 mM, pH 7) at 37 °C for 10 min. Phosphate buffer served as a blank, while Trolox was used as the standard. Following incubation, 25 μL of an AAPH (2,2′-azobis(2-methylpropionamidine) dihydrochloride) solution (150 mM in phosphate buffer) was added. Fluorescence decay was monitored over 90 min at excitation and emission wavelengths of 485 nm and 535 nm, respectively, using a TECAN Infinite F200 PRO microplate reader. Results were expressed as µmol TE per gram of dry plant material, calculated using a Trolox standard curve (3.125–50 µmol/L; R^2^ = 0.9854, y = 38,411x + 6.9683).

#### 2.5.5. Colorimetric Nitric Oxide Scavenging Assay

The nitric oxide (NO) scavenging activity was evaluated using a colorimetric assay using Griess reagent as previously described [21]. Briefly, sodium nitroprusside (SNP, 10 mM) in phosphate buffer solution (PBS) was combined with various concentrations of herbal tea samples (undigested, gastric, or intestinal) (40–500 μg/mL in PBS). A reaction mixture without digested sample was prepared as a negative control. After incubating the mixtures at 37 °C for 2 h, Griess reagent, containing 1% sulfanilamide, 5% phosphoric acid, and 0.1% N-(1-Naphthyl) ethylenediamine dihydrochloride (NED), was added. Following 10 min incubation, the absorbance was measured at 540 nm, using a TECAN Infinite F200 PRO microplate reader. The NO scavenging activity was expressed as IC_50_ values.

### 2.6. Inhibition of the Advanced Glycation End Products (AGEs) Formation

The inhibition of AGEs formation was assessed using the bovine serum albumin (BSA)/D-ribose method, as previously described [7]. In this assay, 40 μL of D-ribose (120 mM), 40 μL of BSA (25 mg/mL), and 20 μL of LV herbal tea or verbascoside digestion samples (undigested, gastric and intestinal) at various concentrations were incubated at 37 °C in a phosphate buffer (50 mM, pH 7.4). Following a 24 h incubation period, AGE fluorescence was measured using a TECAN Infinite F200 PRO microplate reader at excitation and emission wavelengths of 370 nm and 440 nm, respectively. Eight sample concentrations (0.1–12.5 mg/mL) were tested, and antiglycation activity was expressed as IC_50_ values.

### 2.7. HPLC Analysis

HPLC analyses were performed using a Chromaster system (VWR-Hitachi, Radnor, PA, USA) equipped with two L7100 pumps, an L7200 autosampler, an L2450 diode array detector (DAD), and EZ Chrom Elite software version 3.3.2 (Agilent Technologies, Santa Clara, CA, USA). Undigested and digested samples (20 µL) were injected onto a reversed-phase Lichrospher^®^ C18 endcapped column (Merck, Darmstadt, Germany; 125 × 4 mm, 5 µm particle size) maintained at room temperature.

Elution was carried out using water with 1% phosphoric acid (solvent A) and acetonitrile (solvent B) under the following linear gradient conditions (total running time: 50 min): 0–5 min: 5% B, 5–30 min: 5–7% B, 30–45 min: 7–12% B, 45–50 min: 12–40% B. The flow rate was maintained at 1 mL/min, and the detector monitored at a wavelength of 340 nm.

Digestion samples (undigested, gastric, intestinal) were concentrated using a SpeedVac concentrator (SPD121P) with a refrigerated vapor trap (RVT5105, Thermo Fisher Scientific, Waltham, MA, USA) and redissolved in H_2_O to 12.5 mg/mL. Standards were dissolved in H_2_O, except L7DG, which was prepared in H_2_O/DMSO (80:20, *v*/*v*). Compounds were identified by retention time and DAD-UV/Vis spectra (200–400 nm) characteristics in comparison to commercial standards.

Quantitative analysis was carried out using calibration curves based on the UV signal at 340 nm for each standard: caffeic acid, 3.12–125 µg/mL (y = 1475.47x + 3.69; R^2^ = 0.999); L7DG, 20–300 µg/mL (y = 645.48x − 26.18; R^2^ = 0.999); verbascoside, 12.5–100 µg/mL (y = 588.05x + 4.68; R^2^ = 0.999) and isoverbascoside, 12.5–100 µg/mL (y = 445.31x − 0.82; R^2^ = 0.999).

### 2.8. Pre-Column DPPH•-HPLC Evaluation of LV Infusion

DPPH•-HPLC experiments were performed following the method previously described [18]. Specifically, LV intestinal phase solution (12.5 mg/mL) was mixed with DPPH• solutions of various concentrations (0.5 mM, 1 mM and 2 mM in methanol) at a 1:2 (*v*/*v*) ratio. The mixtures were vortexed and incubated at room temperature for 30 min before being analysed by HPLC as previously described. Control consisted of solution of intestinal phase (12.5 mg/mL) mixed with methanol in a 1:2 (*v*/*v*) ratio and processed in the same manner as the DPPH•-treated samples. The percentage of remaining constituents was determined by calculating the ratio of the peak area of each constituent after reaction with DPPH• to the peak area of the constituent in the control sample.

### 2.9. Pre-Column MGO-HPLC Evaluation of LV Infusion

MGO-HPLC experiments were conducted following a previously published method [15] with slight modifications. LV intestinal phase solution (12.5 mg/mL) was mixed with MGO solutions at various concentrations (0.5 M, 1 M and 2 M in phosphate buffer 50 mM, pH 7.4) in a 1:3 (*v*/*v*) ratio. The mixtures were vortexed and incubated at 37 °C for 1 h before undergoing HPLC analysis, as previously described. A control sample was prepared by mixing intestinal phase solution (12.5 mg/mL) with phosphate buffer in a 1:3 (*v*/*v*) ratio and incubating under the same conditions as the MGO-enriched solutions. The percentage of remaining constituents was determined by calculating the ratio of the peak area of each constituent after reaction with MGO to its corresponding peak area in the control sample.

### 2.10. Statistical Analysis

All analyses were conducted in triplicate. Results are presented as the mean ± standard error of the mean (SEM). Statistical significance was evaluated using one-way ANOVA, followed by Fisher’s Least Significant Difference (LSD) test. A *p*-value ≤ 0.05 was considered statistically significant.

## 3. Results and Discussion

### 3.1. Effect of Simulated Digestion on the Phenolic Content of LV Herbal Tea

Phenolic compounds are bioactive molecules widely distributed in plants, and recognized for their antioxidant properties and beneficial health effects, particularly in preventing cardiovascular, neurodegenerative, and inflammatory diseases [22]. Widely present in food but also in medicinal plants, polyphenols may contribute to human well-being due to their anti-inflammatory and antioxidant properties. Therefore, it is essential to evaluate their fate during digestion steps in order to better assess the bioactive potential of plant extracts such as herbal teas.

**Figure 2:**
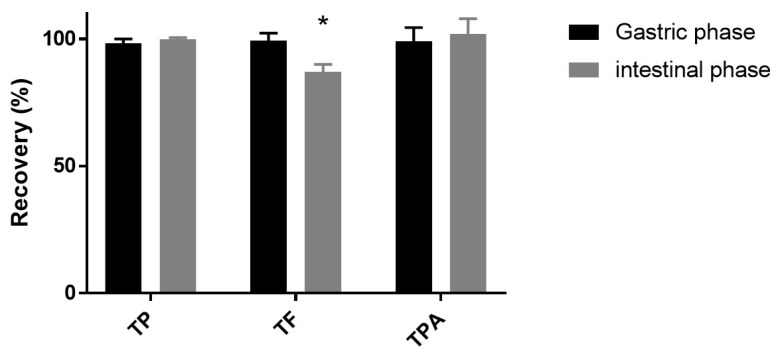
Effect of in vitro simulated digestion of lemon verbena tea on total phenolic content (TP), total flavonoid content (TF) and total phenolic acid (TPA) recovery rate. Data are presented as means ± SEM (n = 3). Results are expressed as percentages with the control (undigested matrix) normalised as 100%. * *p* ≤ 0.05 vs. control.

The impact of in vitro SGID of LV tea on total phenol, flavonoid, and phenolic acid contents is presented in Table 1 and Figure 2. The TPC of LV herbal tea (58.0 ± 0.4 mg GAE/g) aligns with previous studies, which report values ranging from 12 to 71 mg GAE/g of dry plant or commercial batches, in infusion or aqueous extraction [5,23,24]. Our results indicate that in vitro digestion had minimal and non-significant impact on TPC, regardless of the digestion phase (gastric or intestinal).

Flavonoids are a group of polyphenolic compounds particularly abundant in fruits, vegetables, and herbal teas. LV infusion contained 14.6 mg of flavonoids expressed as L7DGE per g of dry plant, resulting in a TFC/TPC ratio of approximately 25%, which is consistent with the values reported by [16] (around 25%). Due to the acidic environment which favors flavonoid stability [12,25], the gastric phase had no significant impact on TFC, with a recovery rate of 99.5%. However, the intestinal phase had a more pronounced and significant effect, with a recovery rate of 87.1%. As noted by Gayoso et al. [12], this decrease may be attributed to the sensitivity of O-glycosylated flavonoids to variations in pH and temperature [25]. However, it may also be attributed to enzymatic transformations involving decarboxylation and dehydroxylation reactions, which simplify the aromatic structures and lead to the formation of metabolites such as phenolic acids [26].

Phenolic acids are the predominant phenolic compounds in LV herbal tea, representing approximately 70% of TPC with 39.0 mg VE/g of dry plant, in agreement with commonly reported values for LV infusions or aqueous extracts [1,27]. TPAC was not significantly affected throughout the different digestion phases. Although some studies have reported loss of phenolic compounds, such as cinnamic acid derivatives, during the gastric phase [28,29], most have shown that these compounds are stable at this stage. This could be due to the acidic pH of the gastric phase that can protect these compounds [7,30]. Conversely, incubation in a neutral or slightly basic intestinal environment, coupled with the presence of pancreatin, is regularly associated with losses [7,12,29,30]. In this study, stability of phenolic acids observed during the intestinal phase may be attributed to the degradation of the major compound, verbascoside, into isoverbascoside and caffeic acid, thus preserving the ability to react with Arnow reagent [9,31,32].

### 3.2. HPLC Phenolic Profile of LV Herbal Tea

**Figure 3:**
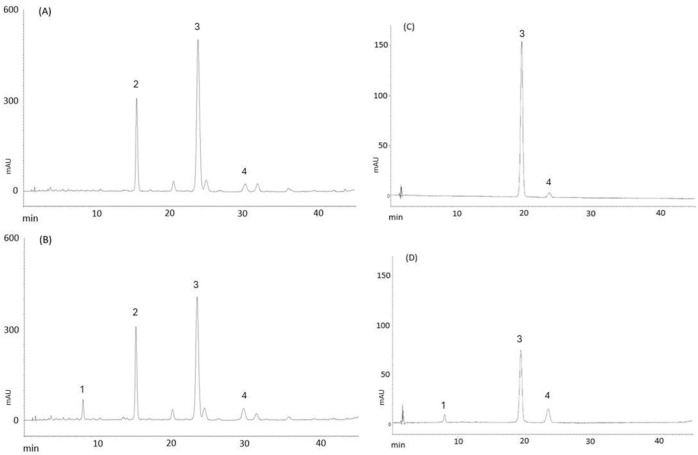
HPLC phenolic profile at 340 nm of lemon verbena tea (**A**,**B**) and verbascoside (**C**,**D**) before and after in vitro simulated digestion. (**A**) Undigested lemon verbena tea, (**B**) lemon verbena tea after intestinal phase, (**C**) Undigested verbascoside, (**D**) verbascoside after intestinal phase. Peaks: (1) caffeic acid; (2) luteolin 7-O-diglucuronide; (3) verbascoside; (4) isoverbascoside.

**Figure 4:**
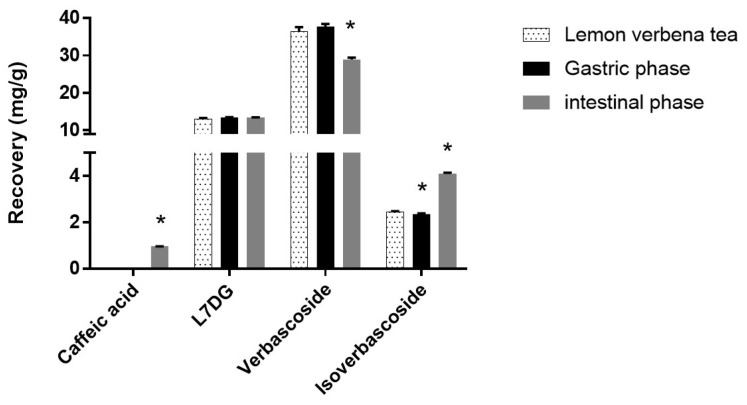
Impact of in vitro gastrointestinal simulated digestion of lemon verbena tea on main polyphenols, luteolin 7-O-diglucuronide (L7DG), verbascoside and isoverbascoside, and main secondary metabolite, caffeic acid, quantified by HPLC. Data are presented as means ± SEM (n = 3). Results are expressed mg/g of dry plant. * *p* ≤ 0.05 vs. control.

The HPLC profile of LV herbal tea, before or after SGID, is shown in Figure 3. We focused more particularly on the three main polyphenols, L7DG, verbascoside and isoverbascoside, as well as on the main secondary metabolite, caffeic acid (Figure 4).

After the gastric phase, no significant decrease in L7DG occurred (13.1 mg/g vs. 13.4 mg/g of dry plant for undigested and gastric samples, respectively; *p* > 0.05). In line with the TFC, this finding confirms the hypothesis that gastric digestion conditions (pepsin and acidity) had no impact on flavonoid stability, as previously reported [7,13,30]. After the intestinal step, no decrease in L7DG was also observed (13.1 mg/g in undigested samples vs. 13.4 mg/g in intestinal samples). The stability of L7DG, as quantified by HPLC, contrasts with the decrease observed for TFC, aligning with the general trend reported after gastrointestinal digestion [7,13,30,33]. The slight decrease in TFC observed in our study may be attributed to the degradation of minor herbal tea flavonoids not quantified in HPLC.

Verbascoside content was not significantly affected by gastric stage (36.5 mg/g and 37.7 mg/g of dry plant before and after gastric stage, respectively; *p* > 0.05). However, a significant decrease (*p* ≤ 0.05) was observed after the intestinal step, reducing its content to 28.9 mg/g of dry plant (−20.8%). A reduction in verbascoside content during the transition from gastric to intestinal conditions has also been previously described [31,34]. Mihailovic et al. [31] described the marked instability of verbascoside during intestinal digestion, suggesting that its degradation during the small intestinal phase may be related to the higher pH conditions of this phase or to enzymatic hydrolysis. Isoverbascoside content, low in the undigested matrix, remained stable during gastric digestion (2.5 vs. 2.4 mg/g of dry plant, respectively) but nearly doubled during the intestinal phase (4.10 mg/g of dry plant). Similarly, caffeic acid, only present in trace amounts in the undigested and gastric matrices, became largely quantifiable in the intestinal phase (1.0 mg/g of dry plant). These findings align with those of D’Imperio et al. [9], who reported that verbascoside remains stable at pH 3 but is significantly degraded at pH 7, leading to the simultaneous formation of its isomer, isoverbascoside. As verbascoside is an ester of caffeic acid, the observed increase in caffeic acid concentration during the intestinal phase is likely related to its degradation [31].

### 3.3. Effect of Simulated Digestion on Antiglycoxidant Capacity of LV Herbal Tea

It is now well documented that chronic diseases such as cardiovascular diseases, cancer, chronic inflammation, neurodegenerative diseases are linked to oxidative stress and that consumption of polyphenols could help reduce these risks [22]. The antioxidant potential of polyphenols operates through several well-known mechanisms, including free radical scavenging via hydrogen transfer, metal ion chelation, and oxygen reduction [35]. As a result, assessing the antioxidant capacity of a plant sample is complex due to the diversity of oxidation processes and the complexity of its matrix. In this study, we employed complementary methods to evaluate various antioxidant mechanisms: free radical scavenging (DPPH•, ABTS•+ and NO° scavenging assays), metal ion reduction (FRAP assay), and protection of a target exposed to free radicals (ORAC assay) [35,36].

Before SGID, results of the antioxidant activity evaluation (Table 2) are consistent with those generally reported [7,37]. After SGID, DPPH• scavenging capacity was unaffected, with recovery in gastric and intestinal samples of 103% and 101%, respectively, compared to undigested samples (*p* > 0.05). Similarly, the ferric reducing ability was not significantly affected by digestion, with recovery rates of 103% and 102% in gastric and intestinal samples, respectively (*p* > 0.05). Moreover, NO° scavenging activity remained stable in the gastric phase, while there was a slight but not significant decrease (*p* > 0.05) of IC50 in the intestinal phase compared to the undigested sample. In contrast, the TEAC assay revealed a significant increase (115%; *p* ≤ 0.05) in antioxidant capacity during the intestinal phase compared to both undigested and gastric phases (102%). Furthermore, ORAC was significantly enhanced after intestinal digestion, with recoveries of 104% and 121% for gastric and intestinal samples, respectively. This study thus demonstrated that antioxidant ability of LV herbal tea polyphenols remained stable or even improved after SGID. This finding is consistent with results reported for other herbal teas [7,37,38]. Despite a slight reduction in TFC, preservation of antioxidant activity may be attributed to matrix effects, as well as to transformation of some polyphenols into other antioxidant compounds that could contribute to this observation. Our results are consistent with those reported by Pineda-Vadillo et al. [39], who demonstrated that food matrices can protect polyphenols from degradation during the intestinal phase, allowing bound compounds to exert biological effects at the colonic level following microbial metabolism.

AGEs are formed through a non-enzymatic reaction between the amino groups of proteins and the carbonyl groups of reducing sugars, a process known as Maillard reaction. The accumulation of AGEs is a key mechanism in cellular aging, but also contributing to oxidative stress and inflammation, leading to chronic diseases such as diabetes, atherosclerosis, neuropathy, and chronic kidney disease. Preventing AGEs formation through specific inhibitors could offer protection and help delay the onset of these disorders and their complications [40]. Both in vitro and in vivo studies have demonstrated the ability of polyphenols to inhibit AGE formation, in connection with their antioxidant properties. Therefore, identifying plant extracts with antiglycative activity represents a significant health interest [3].

SGID slightly improved the antiglycative activity of LV tea (Table 2). However, this activity remained lower than that of the reference compound aminoguanidine (IC50: 250 µg/mL). Given that phenolic acids represent around 70% of total LV tea polyphenols and that verbascoside is the dominant compound among them, it is likely that verbascoside was the primary contributor to this antiglycative activity. Caffeic acid and its derivatives are known for strong antiglycation effects, even surpassing aminoguanidine [41,42]. The observed increase in caffeic acid release during the intestinal phase, along with the isomerization of verbascoside to isoverbascoside, likely contributes to the stability in antiglycative efficacy post-digestion. Flavonoids are also well known for their strong antiglycative activity, with luteolin being among the most effective compounds in this regard [3]. However, their glycosylated forms, such as L7DG, tend to exhibit lower activity compared to their aglycone counterparts [7,40]. The remarkable stability of L7DG during the intestinal phase of digestion may thus contribute to the stable antiglycative activity of LV herbal tea.

### 3.4. Effect of Simulated Digestion on Verbascoside Standard

Verbascoside is commonly found in various plants, and known for its wide range of biological activities, including strong antioxidant and anti-inflammatory properties [31]. Since it is the major compound in LV herbal tea, we have also submitted it to SGID, under identical conditions.

HPLC analysis (Figure 3) has shown more pronounced changes compared to those observed after digestion of LV herbal tea. While verbascoside remained stable in the gastric phase, a marked and significant decrease in its content (−45%, *p* ≤ 0.05) was observed in the intestinal matrix, in accordance with D’Imperio et al. [9]. Moreover, in the intestinal phase, a substantial increase (+441%) in isoverbascoside level (183 mg/g vs. 43 mg/g in undigested phase, *p* ≤ 0.05) and appearance of caffeic acid were observed, aligning with previous reports [31,34]. Caffeic acid, initially undetectable, reached a content of 12 mg/g (Table 3).

DPPH• scavenging activity of verbascoside (Table 4) was stable during the gastric phase (99.4% recovery), while a significant decrease (*p* ≤ 0.05) occurred in the intestinal phase (89.3% recovery), unlike observed for the complex matrix that is LV tea. The presence of other free radical scavengers in the herbal tea, assuming a matrix effect, could partially explain this difference. Moreover, TEAC values remained stable throughout the digestion process (recovery rates of 101% and 107% in the gastric and intestinal phases, respectively).

Before digestion, verbascoside exhibited a remarkably high antiglycative potential, with an IC50 of 128 µg/mL, higher than the reference compound, aminoguanidine (IC50: 250 µg/mL). Following SGID, this effect is further amplified, as the IC50 decreased to 63.4 µg/mL, indicating a 50% increase in efficacy compared to the undigested phase (Table 4). This substantial improvement suggests that digestion enhances the antiglycative potential of verbascoside, likely through structural modifications and significant activity of created compounds.

### 3.5. DPPH• Spiking Experiments for Detection of Main Radical Scavengers in Intestinal Samples from LV Herbal Tea

The DPPH• spiking assay has been recognized as a rapid and reliable method for detecting antioxidant constituents in complex biological matrices [18]. This approach relies on the principle that radical scavenging compounds undergo structural modifications upon reacting with radical species [35,36]. As a result, the peak area of bioactive constituents decreases in the HPLC chromatogram of DPPH•-pretreated extracts, allowing for the identification and quantification of antioxidant compounds within the sample.

Most polyphenols are mainly absorbed in the small intestine and, to a lesser extent, in the colon [43]. It was therefore important to evaluate, among the potential LV bioavailable compounds present in intestinal samples, those with ability to scavenge DPPH• radicals.

**Figure 5:**
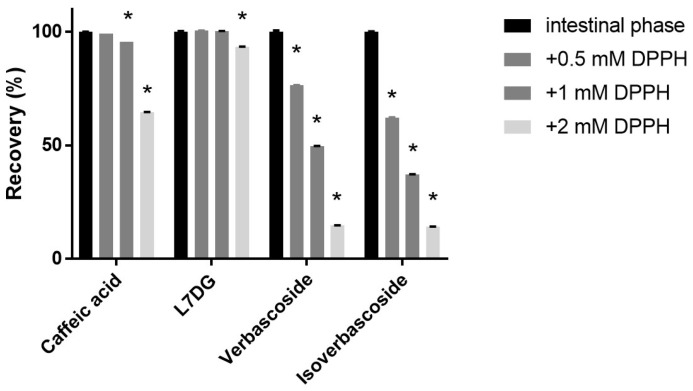
Percentage of remaining compounds after incubation of lemon verbena herbal tea (intestinal phase) with various concentrations of DPPH• (0.5, 1 and 2 mM). L7DG: luteolin 7-O-diglucuronide. Data are presented as means ± SEM (n = 3). * *p* ≤ 0.05 vs. control (intestinal phase).

Spiking experiments (Figure 5) with 0.5 mM DPPH• radical demonstrated stability of L7DG and caffeic acid (recovery rates of 100% and 99.0%, respectively). However, in similar conditions, a significant decrease was observed for verbascoside and its isomer, isoverbascoside (recovery rates of 76.4% and 62.2%, respectively; *p* ≤ 0.05). Use of a higher DPPH• radical concentration (1 mM) confirmed the stability of L7DG (100% recovery) but revealed a slight but significant decrease in caffeic acid peak area (95.5% recovery; *p* ≤ 0.05). In contrast, verbascoside and isoverbascoside peak areas were highly reduced (recovery rates of 49.6% and 37.1%, respectively; *p* ≤ 0.05), highlighting their strong radical scavenging activity. At an even higher radical concentration (2 mM), all compounds of interest showed a significant decrease. Only a slight reduction was observed for L7DG (93.3% recovery), whereas caffeic acid showed a more pronounced decline (64.6% recovery), and verbascoside and isoverbascoside were nearly depleted (recovery rates of 14.7% and 14.2%, respectively; *p* ≤ 0.05).

These findings suggest that main LV phenolics exhibit radical scavenging potential that varies considerably depending on their structure. Despite the presence of two hydroxyl groups on the B ring, a characteristic recognized as responsible for strong antioxidant potential [10], L7DG appears to have low trapping activity. In contrast, verbascoside and its isomer, isoverbascoside, emerge as the most reactive compounds in the herbal tea, likely due to the presence of two catechol groups. Caffeic acid, containing a single catechol group, is less effective than verbascoside and isoverbascoside. Notably, these results align with previous studies on the relationship between chemical structure and DPPH• radical scavenging activity of phenolic acids and flavonoids [44,45].

### 3.6. MGO Spiking Experiments for Detection of Main Dicarbonyl Trapping Constituents in Intestinal Samples from LV Herbal Tea

**Figure 6:**
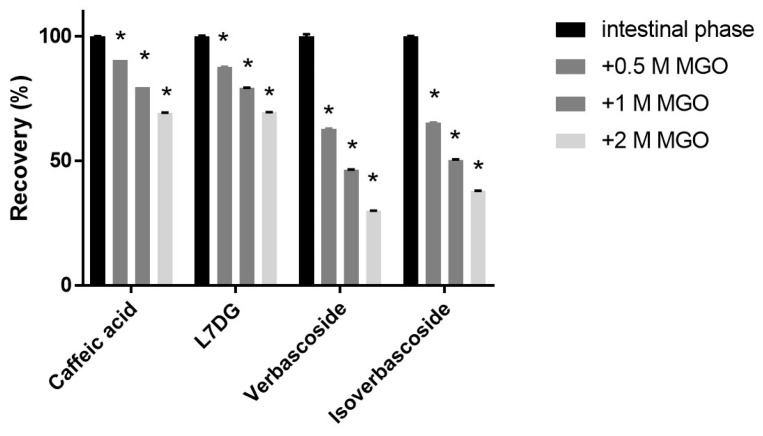
Percentage of remaining compounds after incubation of lemon verbena herbal tea (intestinal phase) with various concentrations of MGO (0.5, 1 and 2 M). L7DG: luteolin 7-O-diglucuronide. Data are presented as means ± SEM (n = 3). * *p* ≤ 0.05 vs. control (intestinal phase).

Direct scavenging of α-dicarbonyl compounds like methylglyoxal (MGO), key contributors to AGE formation, is an effective strategy to mitigate their development [18]. In complex plant extract matrices, with a high abundance of polyphenols, employing a straightforward method to identify the primary MGO-trapping active compounds is essential for understanding the mechanism behind their antiglycative effects [46,47]. As with the DPPH• spiking assay, we focused specifically on the intestinal matrix, given that bioactive compounds are primarily absorbed in the small intestine. This approach allows relevant assessment of the antiglycative potential of LV herbal tea and its key constituents after digestion. As shown in Figure 6, regardless of the MGO concentration incubated with the intestinal matrix, a significant decrease in peak area of all compounds of interest was observed, confirming the strong antiglycative properties of LV herbal tea. At the lowest MGO concentration (0.5 M), only a slight but significant reduction in the area under the curve for caffeic acid and L7DG was noted (recovery rates of 90.5% and 82.8%, respectively) whereas verbascoside and isoverbascoside exhibited a much greater trapping capacity (recovery rates of 63.0% and 65.5%, respectively). Incubation with 1 M MGO followed the same trend, highlighting the lower efficiency of caffeic acid and L7DG compared to verbascoside and isoverbascoside. At the highest concentration (2 M), this difference became even more pronounced. Indeed, while around 80% of caffeic acid and L7DG were recovered, only 30% and 38% of verbascoside and isoverbascoside, respectively, were still present.

It should thus be noted that there is a difference in the MGO trapping capacity among LV tea compounds, which can be attributed to distinct chemical structures. The presence and position of hydroxyl and catechol groups play a crucial role in determining their reactivity with α-dicarbonyl compounds like MGO [40]. Flavonoids are widely recognized for their strong antiglycative activity [3]. Since the primary site for carbonyl trapping is an aromatic ring with two hydroxyl groups, luteolin, characterized by 5,7-dihydroxyl substitutions on its A ring, is considered among the most effective flavonoids [18]. However, glycosylation generally reduces the antiglycative potential of flavones compared to their aglycone forms [40]. In the case of L7DG, its glucuronidation on C7 likely accounts for this diminished activity, leaving only the catechol group on the B ring available for carbonyl trapping. Liu et al. [42] further reported that the antiprotein glycation activities of verbascoside and its isomer, isoverbascoside, stem from their ability to trap reactive carbonyl species via their constituent moieties, caffeic acid and 3,4-dihydroxyphenylethanol. The presence of catechol groups in both these components results in a stronger trapping effect than for caffeic acid alone. This could explain the substantial decrease in verbascoside and isoverbascoside peak areas observed for all MGO concentrations used in our study.

## 4. Conclusions

This study shows that LV tea phenolic compounds, including flavonoids and phenolic acids, remain largely stable during in vitro digestion, with maintained or slightly improved antioxidant activity. Although verbascoside is partly degraded in the intestinal phase, its potential conversion into isoverbascoside and caffeic acid suggests the formation of bioactive derivatives that may contribute to overall activity.

These findings support the potential use of LV tea as a functional beverage with preserved antioxidant and antiglycation properties after digestion, and open perspectives for its valorization in nutraceutical and health-oriented applications.

## Figures and Tables

**Table 1:** Impact of SGID procedure on total phenolic, total flavonoid and total phenolic acid contents of lemon verbena tea.

Assay	Undigested Matrix	Gastric Phase	Intestinal Phase
Total phenolic content (mg GAE/g of dry plant)	58.0 ± 0.4 ^a^	56.9 ± 0.6 ^a^	57.9 ± 0.3 ^a^
Total flavonoid content (mg L7DGE/g of dry plant)	14.6 ± 0.4 ^a^	14.4 ± 0.1 ^a^	12.6 ± 0.2 ^b^
Total phenolic acid content (mg VE/g of dry plant)	39.0 ± 2.1 ^a^	38.6 ± 6.8 ^a^	39.8 ± 1.4 ^a^

Results are expressed as mean values ± SEM (n = 3). Values in the same row sharing identical superscript are not significantly different from each other (*p* > 0.05). SGID: simulated gastrointestinal digestion, GAE: gallic acid equivalents, L7DGE: luteolin-7-diglucuronide equivalents, VE: verbascoside equivalents.

**Table 2:** Impact of SGID on antiglycoxidant activities of lemon verbena tea.

Assay	Undigested Matrix	Gastric Phase	Intestinal Phase
DPPH• (µmol of TE/g of dry plant)	268 ± 5	275 ± 3 ^a^	272 ± 6 ^a^
TEAC (µmol of TE/g of dry plant)	364 ± 7 ^a^	368 ± 7 ^a^	419 ± 7 ^b^
FRAP (µmol of TE/g of dry plant)	551 ± 7 ^a^	565 ± 2 ^a^	564 ± 4 ^a^
ORAC (µmol of TE/g of dry plant)	1568 ± 43 ^a^	1627 ± 9 ^a^	1880 ± 72 ^b^
NO° scavenging activity IC50 (µg/mL)	200 ± 15 ^a^	204 ± 2 ^a^	193 ± 12 ^a^
Antiglycation activity IC50 (µg/mL)	514 ± 14 ^a^	520 ± 79 ^a^	374 ± 26 ^b^

Results are expressed as mean values ± SEM (n = 3). Values in the same row sharing identical superscript are not significantly different from each other (*p* > 0.05). SGID: simulated gastrointestinal digestion, TE: Trolox equivalent.

**Table 3:** Impact of SGID of verbascoside standard on caffeic acid, verbascoside and isoverbascoside contents quantified by HPLC.

Compound	Undigested Matrix	Gastric Phase	Intestinal Phase
Caffeic acid (mg/g)	ND ^a^	ND ^a^	12.0 ± 0.9
Verbascoside (mg/g)	1030 ± 18 ^a^	1056 ± 15 ^a^	564 ± 14 ^b^
Isoverbascoside (mg/g)	42.6 ± 7.5 ^a^	44.1 ± 4.0 ^a^	183 ± 4 ^b^

Results are expressed as mean values ± SEM (n = 3). Values in the same row sharing identical superscript are not significantly different from each other (*p* > 0.05). SGID: simulated gastrointestinal digestion, ND: Not Detectable.

**Table 4:** Impact of SGID procedure on antiglycoxidant activities of verbascoside.

Assay	Undigested Matrix	Gastric Phase	Intestinal Phase
DPPH• (µmol of TE/g)	4064 ± 51 ^a^	4038 ± 19 ^a^	3626 ± 32 ^b^
TEAC (µmol of TE/g)	4337 ± 142 ^a^	4387 ± 23 ^a^	4627 ± 136 ^a^
Antiglycation activity IC50 (µg/mL)	129 ± 3 ^a^	120 ± 5 ^a^	63.4 ± 2.7 ^b^

Results are expressed as mean values ± SEM (n = 3). Values in the same row sharing identical superscript are not significantly different from each other (*p* > 0.05). SGID: simulated gastrointestinal digestion, TE: Trolox equivalent.

## Data Availability

The original contributions presented in this study are included in the article. Further inquiries can be directed to the corresponding author.

## References

[1] Polumackanycz M., Petropoulos S.A., Añibarro-Ortega M., Pinela J., Barros L., Plenis A., Viapiana A. (2022). Chemical Composition and Antioxidant Properties of Common and Lemon Verbena. Antioxidants.

[2] Atoui A. (2005). Tea and Herbal Infusions: Their Antioxidant Activity and Phenolic Profile. Food Chem..

[3] Yeh W.-J., Hsia S.-M., Lee W.-H., Wu C.-H. (2017). Polyphenols with Antiglycation Activity and Mechanisms of Action: A Review of Recent Findings. J. Food Drug Anal..

[4] Abderrahim F., Estrella S., Susín C., Arribas S., González M., Condezo L. (2011). The Antioxidant Activity and Thermal Stability of Lemon Verbena (*Aloysia triphylla*) Infusion. J. Med. Food.

[5] Peixoto J.A.B., Álvarez-Rivera G., Costa A.S.G., Machado S., Cifuentes A., Ibáñez E., Oliveira M.B.P.P., Alves R.C. (2023). Contribution of Phenolics and Free Amino Acids on the Antioxidant Profile of Commercial Lemon Verbena Infusions. Antioxidants.

[6] Funes L., Fernández-Arroyo S., Laporta O., Pons A., Roche E., Segura-Carretero A., Fernández-Gutiérrez A., Micol V. (2009). Correlation between Plasma Antioxidant Capacity and Verbascoside Levels in Rats after Oral Administration of Lemon Verbena Extract. Food Chem..

[7] Fraisse D., Bred A., Lagarde A., Felgines C. (2025). Impact of Simulated Gastrointestinal Conditions on Polyphenol Stability and Antiglycoxidant Potential of Sage (*Salvia officinalis*) Infusion. S. Afr. J. Bot..

[8] Raghunath S., Budaraju S., Gharibzahedi S.M.T., Koubaa M., Roohinejad S., Mallikarjunan K. (2023). Processing Technologies for the Extraction of Value-Added Bioactive Compounds from Tea. Food Eng. Rev..

[9] D’Imperio M., Cardinali A., D’Antuono I., Linsalata V., Minervini F., Redan B.W., Ferruzzi M.G. (2014). Stability–Activity of Verbascoside, a Known Antioxidant Compound, at Different pH Conditions. Food Res. Int..

[10] Sánchez-Marzo N., Lozano-Sánchez J., Cádiz-Gurrea M.d.l.L., Herranz-López M., Micol V., Segura-Carretero A. (2019). Relationships Between Chemical Structure and Antioxidant Activity of Isolated Phytocompounds from Lemon Verbena. Antioxidants.

[11] Minekus M., Alminger M., Alvito P., Ballance S., Bohn T., Bourlieu C., Carrière F., Boutrou R., Corredig M., Dupont D. (2014). A Standardised Static in Vitro Digestion Method Suitable for Food—An International Consensus. Food Funct..

[12] Gayoso L., Claerbout A.-S., Calvo M.I., Cavero R.Y., Astiasarán I., Ansorena D. (2016). Bioaccessibility of Rutin, Caffeic Acid and Rosmarinic Acid: Influence of the in Vitro Gastrointestinal Digestion Models. J. Funct. Foods.

[13] Tagliazucchi D., Verzelloni E., Bertolini D., Conte A. (2010). In Vitro Bio-Accessibility and Antioxidant Activity of Grape Polyphenols. Food Chem..

[14] Cardinali A., Linsalata V., Lattanzio V., Farruzzi M. (2011). Verbascosides from Olive Mill Waste Water: Assessment of Their Bioaccessibility and Intestinal Uptake Using an in Vitro Digestion/Caco-2 Model System. J. Food Sci..

[15] Fraisse D., Bred A., Felgines C., Senejoux F. (2020). Stability and Antiglycoxidant Potential of Bilberry Anthocyanins in Simulated Gastrointestinal Tract Model. Foods.

[16] Carnat A., Carnat A.P., Fraisse D., Lamaison J.L. (1999). The Aromatic and Polyphenolic Composition of Lemon Verbena Tea. Fitoterapia.

[17] Gawlik-Dziki U. (2012). Dietary Spices as a Natural Effectors of Lipoxygenase, Xanthine Oxidase, Peroxidase and Antioxidant Agents. LWT-Food Sci. Technol..

[18] Fraisse D., Bred A., Felgines C., Senejoux F. (2020). Screening and Characterization of Antiglycoxidant Anthocyanins from *Vaccinium myrtillus* Fruit Using DPPH and Methylglyoxal Pre-Column HPLC Assays. Antioxidants.

[19] Katalinić V., Generalić I., Skroza D., Ljubenkov I., Teskera A., Konta I., Boban M. (2009). Insight in the Phenolic Composition and Antioxidative Properties of *Vitis vinifera* Leaves Extracts. Croat. J. Food Sci. Technol..

[20] Ndoye S.F., Fraisse D., Akendengué B., Dioum M.D., Gueye R.S., Sall C., Seck I., Felgines C., Seck M., Senejoux F. (2018). Antioxidant and Antiglycation Properties of Two Mango (*Mangifera indica* L.) Cultivars from Senegal. Asian Pac. J. Trop. Biomed..

[21] Fraisse D., Degerine-Roussel A., Bred A., Ndoye S.F., Vivier M., Felgines C., Senejoux F. (2018). A Novel HPLC Method for Direct Detection of Nitric Oxide Scavengers from Complex Plant Matrices and Its Application to *Aloysia triphylla* Leaves. Molecules.

[22] Rana A., Samtiya M., Dhewa T., Mishra V., Aluko R.E. (2022). Health Benefits of Polyphenols: A Concise Review. J. Food Biochem..

[23] Touati Z., Guemghar M., Bedjaoui K., Djerrada N.E., Djaoud K., Adjeroud N., Madani K., Boulekbache-Makhlouf L.E. (2021). Optimization of the Microwave Assisted Extraction and Biological Activities of Polyphenols from Lemon Verbena Leaves. Ann. Univ. Dunarea Jos Galati Fascicle VI-Food Technol..

[24] Younes Allam A., Farouk Elsadek M., S. Al-Numair K., Abdelrasoul A. Badr A., Singh S., Rafat Elkabary M. (2024). Antioxidant and Antibacterial Effect of Lemon Verbena Leaves’ (*Lippia citriodora*) Extract as a Natural Preservative on Refrigerated Meat Patties during Storage. Ital. J. Food Sci..

[25] Xie L., Deng Z., Zhang J., Dong H., Wang W., Xing B., Liu X. (2022). Comparison of Flavonoid O-Glycoside, C-Glycoside and Their Aglycones on Antioxidant Capacity and Metabolism during In Vitro Digestion and In Vivo. Foods.

[26] Morais I.S.d.S.S., Pinheiro L.M.B., Santos F.P., Lima M.d.S., Santos K.M.O.d., Albuquerque C.L.C.d., Cardarelli H.R. (2025). Extraction Processes, Bioaccessibility, Antioxidant Capacity, and Potential Prebiotic Effect of Co-Product Extracts From Fruits of the Spondias Genus. J. Food Sci..

[27] Bilia A.R., Giomi M., Innocenti M., Gallori S., Vincieri F.F. (2008). HPLC–DAD–ESI–MS Analysis of the Constituents of Aqueous Preparations of Verbena and Lemon Verbena and Evaluation of the Antioxidant Activity. J. Pharm. Biomed. Anal..

[28] Siracusa L., Kulisic-Bilusic T., Politeo O., Krause I., Dejanovic B., Ruberto G. (2011). Phenolic Composition and Antioxidant Activity of Aqueous Infusions from *Capparis spinosa* L. and *Crithmum maritimum* L. before and after Submission to a Two-Step in Vitro Digestion Model. J. Agric. Food Chem..

[29] Vallejo F., Gil-Izquierdo A., Pérez-Vicente A., García-Viguera C. (2004). In Vitro Gastrointestinal Digestion Study of Broccoli Inflorescence Phenolic Compounds, Glucosinolates, and Vitamin C. J. Agric. Food Chem..

[30] Bermúdez-Soto M.-J., Tomás-Barberán F.-A., García-Conesa M.-T. (2007). Stability of Polyphenols in Chokeberry (*Aronia melanocarpa*) Subjected to in Vitro Gastric and Pancreatic Digestion. Food Chem..

[31] Mihailović V., Kreft S., Benković E.T., Ivanović N., Stanković M.S. (2016). Chemical Profile, Antioxidant Activity and Stability in Stimulated Gastrointestinal Tract Model System of Three Verbascum Species. Ind. Crops Prod..

[32] Santoro A., Bianco G., Picerno P., Aquino R.P., Autore G., Marzocco S., Gazzerro P., Lioi M.B., Bifulco M. (2008). Verminoside- and Verbascoside-Induced Genotoxicity on Human Lymphocytes: Involvement of PARP-1 and P53 Proteins. Toxicol. Lett..

[33] Gonçalves G.A., Corrêa R.C.G., Barros L., Dias M.I., Calhelha R.C., Correa V.G., Bracht A., Peralta R.M., Ferreira I.C.F.R. (2019). Effects of in Vitro Gastrointestinal Digestion and Colonic Fermentation on a Rosemary (*Rosmarinus officinalis* L.) Extract Rich in Rosmarinic Acid. Food Chem..

[34] Jiang Y., Mao S., Huang W., Lu B., Cai Z., Zhou F., Li M., Lou T., Zhao Y. (2016). Phenylethanoid Glycoside Profiles and Antioxidant Activities of *Osmanthus fragrans* Lour. Flowers by UPLC/PDA/MS and Simulated Digestion Model. J. Agric. Food Chem..

[35] Shahidi F., Zhong Y. (2015). Measurement of Antioxidant Activity. J. Funct. Foods.

[36] Munteanu I.G., Apetrei C. (2021). Analytical Methods Used in Determining Antioxidant Activity: A Review. Int. J. Mol. Sci..

[37] Qin W., Hu F., Hui S. (2025). Comparative Study of Total Polyphenol Content and Antioxidant Activity of Yomogi Tea and Green Tea during Simulated In Vitro Gastrointestinal Digestion. ACS Food Sci. Technol..

[38] Olennikov D.N., Kashchenko N.I., Chirikova N.K., Vasil’eva A.G., Gadimli A.I., Isaev J.I., Vennos C. (2019). Caffeoylquinic Acids and Flavonoids of Fringed Sagewort (*Artemisia frigida* Willd.): HPLC-DAD-ESI-QQQ-MS Profile, HPLC-DAD Quantification, in Vitro Digestion Stability, and Antioxidant Capacity. Antioxidants.

[39] Pineda-Vadillo C., Nau F., Dubiard C.G., Cheynier V., Meudec E., Sanz-Buenhombre M., Guadarrama A., Tóth T., Csavajda É., Hingyi H. (2016). In Vitro Digestion of Dairy and Egg Products Enriched with Grape Extracts: Effect of the Food Matrix on Polyphenol Bioaccessibility and Antioxidant Activity. Food Res. Int..

[40] Grzegorczyk-Karolak I., Gołąb K., Gburek J., Wysokińska H., Matkowski A. (2016). Inhibition of Advanced Glycation End-Product Formation and Antioxidant Activity by Extracts and Polyphenols from *Scutellaria alpina* L. and *S. altissima* L.. Molecules.

[41] Khan M., Liu H., Wang J., Sun B. (2020). Inhibitory Effect of Phenolic Compounds and Plant Extracts on the Formation of Advance Glycation End Products: A Comprehensive Review. Food Res. Int..

[42] Liu Y.-H., Lu Y.-L., Han C.-H., Hou W.-C. (2013). Inhibitory Activities of Acteoside, Isoacteoside, and Its Structural Constituents against Protein Glycation in Vitro. Bot. Stud..

[43] Scalbert A., Morand C., Manach C., Rémésy C. (2002). Absorption and Metabolism of Polyphenols in the Gut and Impact on Health. Biomed. Pharmacother. Biomed. Pharmacother..

[44] Jing P., Zhao S.-J., Jian W.-J., Qian B.-J., Dong Y., Pang J. (2012). Quantitative Studies on Structure-DPPH• Scavenging Activity Relationships of Food Phenolic Acids. Molecules.

[45] Yamauchi M., Kitamura Y., Nagano H., Kawatsu J., Gotoh H. (2024). DPPH Measurements and Structure—Activity Relationship Studies on the Antioxidant Capacity of Phenols. Antioxidants.

[46] Ndalane R., Ntuli S., Oberholzer H., Bester M., Serem J. (2026). The Therapeutic Potential of Polyphenols for Targeting Methylglyoxal and the Associated Glycation Pathways: A Review. Adv. Redox Res..

[47] Lee S.M., Zheng L.W., Jung Y., Hwang G.-S., Kim Y.-S. (2020). Effects of Hydroxycinnamic Acids on the Reduction of Furan and α-Dicarbonyl Compounds. Food Chem..

